# An integrated bulk and single-cell transcriptomic analysis reveals stemness-driven immune regulation and therapeutic vulnerability in colorectal cancer

**DOI:** 10.7150/jca.132694

**Published:** 2026-03-25

**Authors:** I-Hung Chen, Kai-Fu Chang, Chien-Cheng Chao, Chung-Hsien Lin, Chih-Hsuan Chang, Ching-Chung Ko, Hui-Ru Lin, Chi-Jen Wu, Chien-Han Yuan, Sachin Kumar, Dahlak Daniel Solomon, Do Thi Minh Xuan, Neethu Palekkode, Ayman Fathima, Yung-Kuo Lee, Chung-Bao Hsieh, Yuen-Jung Wu

**Affiliations:** 1 Department of Internal Medicine, Tri-Service General hospital, National Defense Medical University, Taipei City, Taiwan.; 2 Institute of Medical Science and Technology, National Sun Yat-Sen University, Kaohsiung 80424, Taiwan.; 3 Department of Internal Medicine, Kaohsiung Armed Forces General Hospital, National Defense Medical University, Kaohsiung 80284, Taiwan.; 4 Medical Laboratory, Medical Education and Research Center, Kaohsiung Armed Forces General Hospital, National Defense Medical University, Kaohsiung 80284, Taiwan.; 5 Division of Experimental Surgery Center, Department of Surgery, Tri-Service General Hospital, National Defense Medical Center, Taipei, Taiwan.; 6 Department of Medical Imaging, Chi-Mei Medical Center, Tainan, 710402, Taiwan.; 7 Department of Health and Nutrition, Chia Nan University of Pharmacy and Science, Tainan, 71710, Taiwan.; 8 School of Medicine, College of Medicine, National Sun Yat-Sen University, Kaohsiung, 80424, Taiwan.; 9 Nursing Department, Kaohsiung Armed Forces General Hospital, National Defense Medical University, Kaohsiung, 80284, Taiwan.; 10 College of Nursing, Kaohsiung Medical University, Kaohsiung, 80708, Taiwan.; 11 Department of Otolaryngology, Kaohsiung Armed Forces General Hospital, National Defense Medical University, Kaohsiung, 80284, Taiwan.; 12 Department of Otolaryngology, National Defense Medical Center, Taipei, 11490, Taiwan; 13 PhD Program for Cancer Molecular Biology and Drug Discovery, College of Medical Science and Technology, Taipei Medical University, Taipei, 11031, Taiwan.; 14 Graduate Institute of Cancer Biology and Drug Discovery, College of Medical Science and Technology, Taipei Medical University, Taipei, 11031, Taiwan.; 15 Faculty of Applied Sciences and Biotechnology, Shoolini University of Biotechnology and Management Sciences, Himachal Pradesh, 173229, India.; 16 Yogananda School of AI Computers and Data Sciences, Shoolini University, Solan, 173229, India.; 17 Faculty of Pharmacy, Van Lang University, 69/68 Dang Thuy Tram Street, Binh Loi Trung Ward, Ho Chi Minh City, 70000, Vietnam; 18 Department of Biotechnology, Mother Teresa Women's University, Kodaikanal, Tamil Nadu, 624101, India.; 19 Computer Engineering with specialization in Artificial Intelligence and Machine Learning, Presidency University, Yelahanka, Bengaluru, 560064 India.; 20 School of Medicine, National Defense Medical University, Taipei, 11490, Taiwan.; 21 Taipei City Hospital Zhongxing Branch, Taipei City, 103212, Taiwan.; 22 Department of Surgery, Kaohsiung Armed Forces General Hospital, National Defense Medical University, Kaohsiung, 80284, Taiwan.

**Keywords:** colorectal cancer, tumor stemness, immune microenvironment, immunotherapy, single-cell RNA sequencing, prognostic model

## Abstract

Tumor stemness is increasingly recognized as a key contributor to tumor heterogeneity, immune regulation, and therapeutic resistance in colorectal cancer (CRC). In this study, we developed a stemness-based risk model using bulk transcriptomic data from The Cancer Genome Atlas and evaluated its prognostic and therapeutic relevance through integrative analyses. The proposed risk score robustly stratified patients into distinct prognostic groups and remained an independent predictor of overall survival after adjustment for clinicopathological variables. Stemness-high tumors exhibited altered immune infiltration patterns and coordinated upregulation of immune checkpoint-related genes. Although the association between stemness score and immune evasion potential was modest, its clinical relevance was supported by validation in independent immunotherapy-treated cohorts, where low-risk patients demonstrated improved survival and higher response rates. Single-cell RNA sequencing (scRNA-seq) analysis further revealed that enhanced stemness and dedifferentiation were predominantly localized within malignant epithelial cells. Together, these findings establish tumor stemness as a central determinant of prognosis, immune regulation, and therapeutic vulnerability in CRC.

## Introduction

Colorectal cancer (CRC) remains one of the leading causes of cancer-related morbidity and mortality worldwide, despite substantial advances in surgical techniques, chemotherapy, targeted therapy, and immunotherapy 1-4. However, patient outcomes remain highly heterogeneous, even among individuals with similar clinicopathological features. This heterogeneity reflects the complex biological diversity of colorectal tumors, which arises from genetic, epigenetic, and microenvironmental factors 5-7. Consequently, conventional staging systems alone are insufficient to accurately predict prognosis or therapeutic response, underscoring the need for biologically informed biomarkers that capture intrinsic tumor properties 8, 9.

Tumor stemness, defined as the degree to which cancer cells retain stem cell-like characteristics such as self-renewal, plasticity, and dedifferentiation, has emerged as a critical determinant of tumor aggressiveness and therapeutic resistance 10-13. Accumulating evidence suggests that stemness-associated transcriptional programs contribute to tumor progression, metastasis, and recurrence across multiple cancer types. In CRC, elevated stemness has been linked to poor prognosis and resistance to conventional therapies 14-16. Nevertheless, the prognostic significance of stemness-related gene signatures and their integration into clinically applicable risk stratification models remain incompletely understood 17-19.

Beyond its role in intrinsic tumor biology, stemness is increasingly recognized as a key modulator of the tumor immune microenvironment 20. Stemness-high tumors have been reported to exhibit altered immune cell infiltration patterns, dysregulated immune checkpoint signaling, and enhanced immune evasion capabilities 21-23. These features may critically influence responsiveness to immune checkpoint blockade and other immunotherapeutic strategies 24, 25. However, the relationship between stemness and immunotherapy response in CRC is complex and context-dependent, particularly when assessed using bulk transcriptomic data that obscure cell-type-specific contributions 18, 26, 27.

In this study, we developed a stemness-based prognostic risk model for CRC using bulk transcriptomic data and systematically evaluated its associations with survival outcomes, immune infiltration, immune checkpoint regulation, immunotherapy response, and drug sensitivity. To overcome the limitations of bulk-level analyses, we further integrated scRNA-seq data from paired normal and tumor colorectal tissues to resolve the cellular origins and differentiation states underlying stemness-associated phenotypes 28, 29. By combining bulk and single-cell analyses with external validation in immunotherapy-treated cohorts and protein-level verification, our study provides a comprehensive framework linking stemness, tumor heterogeneity, immune regulation, and therapeutic vulnerability in CRC.

## Methods

### Data acquisition and preprocessing of bulk transcriptomic datasets

Bulk RNA sequencing data and corresponding clinical information for CRC patients were obtained from The Cancer Genome Atlas (TCGA) database. Gene expression data were normalized to transcripts per million values and log2-transformed for downstream analyses 30, 31. Patients with incomplete survival information were excluded. External validation datasets, including GSE38882 for prognostic validation and IMvigor210 and GSE78820 for immunotherapy response analyses, were downloaded from the Gene Expression Omnibus or publicly available repositories 32. Batch effects between datasets were addressed using standard normalization procedures where appropriate 33-35.

### Stemness score calculation and patient stratification

Stemness scores were calculated for each tumor sample based on predefined stemness-associated gene expression patterns using a single-sample scoring approach (specifically, the ssGSEA algorithm). Patients were stratified into high- and low-stemness groups using the median stemness score as the cutoff for differential expression and survival analyses 36-38. For selected analyses, patients were further categorized into high, median, and low stemness groups to assess dose-dependent trends 39.

### Differential expression analysis and functional enrichment

Differentially expressed genes between high- and low-stemness groups were identified using linear modeling with empirical Bayes moderation 40-42. Genes with an absolute log2 fold change above 1.0 and adjusted p values below 0.05 were considered significant. Functional enrichment analyses were performed using Gene Ontology and pathway databases to characterize biological processes associated with stemness-related transcriptional changes 43, 44.

### Construction of the stemness-based prognostic risk model

Univariate Cox proportional hazards regression was applied to identify stemness-associated genes significantly correlated with overall survival. Candidate genes were subsequently subjected to least absolute shrinkage and selection operator Cox regression to construct a parsimonious prognostic model 19. The optimal penalty parameter was determined by cross-validation 45-47. Risk scores were calculated as a weighted sum of gene expression values multiplied by their corresponding regression coefficients, and patients were stratified into high- and low-risk groups based on the median risk score 48.

### Survival analysis and independent prognostic evaluation

Kaplan-Meier survival analysis with log-rank tests was used to compare survival outcomes between risk groups 49. Time-dependent receiver operating characteristic curves were generated to evaluate predictive performance at different time points. Univariate and multivariate Cox regression analyses were conducted to assess the independent prognostic value of the risk score after adjustment for clinicopathological variables, including age, sex, and pathological staging. A nomogram integrating molecular and clinical factors was constructed for comparative performance assessment 50.

### Immune infiltration, immune checkpoint, and immune evasion analyses

Tumor immune cell infiltration was estimated using the CIBERSORT algorithm 51. Differences in immune cell composition between risk groups were evaluated using non-parametric statistical tests. Immune checkpoint gene expression profiles were compared across stemness and risk groups. Stromal, immune, and ESTIMATE scores were calculated to assess overall tumor microenvironment composition. Immune evasion potential was evaluated using Tumor Immune Dysfunction and Exclusion scores, and correlations between stemness scores and TIDE scores were assessed using Spearman correlation analysis 52.

### Immunotherapy response validation

The association between the stemness-based risk model and immunotherapy response was evaluated in IMvigor210 and GSE78820 cohorts. Patients were classified according to reported treatment responses, including complete response, partial response, stable disease, and progressive disease. Survival outcomes, risk score distributions, and response proportions were compared between risk groups to assess predictive relevance in immunotherapy-treated populations 29.

### Drug sensitivity prediction

In silico drug sensitivity analysis was performed by estimating half-maximal inhibitory concentration values for multiple therapeutic agents using established pharmacogenomic modeling approaches 53. Estimated IC50 values were compared between high- and low-risk groups 54-56, to identify differential drug susceptibility patterns associated with stemness-based risk stratification 57.

### Single-cell RNA sequencing analysis

ScRNA-seq data from paired normal and tumor colorectal tissues were obtained from GSE196964. Quality control filtering was applied to remove low-quality cells based on gene count and mitochondrial gene expression thresholds 58, 59. Data were normalized, scaled, and subjected to dimensionality reduction using principal component analysis and uniform manifold approximation and projection. Cell clusters were annotated based on canonical marker genes 60-62. CytoTRACE analysis was performed to infer cellular differentiation states and stemness at single-cell resolution. Functional enrichment analyses were conducted for major cell populations 63, 64.

### Immunohistochemical validation

Protein-level validation of selected stemness-associated signature genes was performed using publicly available immunohistochemical staining data from CRC tissue microarrays 65-67. Representative staining patterns and expression distributions were evaluated to support transcriptomic findings 68.

### Statistical analysis

All statistical analyses were conducted using R (4.4.1) software. Survival analyses were performed using Cox proportional hazards models and Kaplan-Meier methods. Group comparisons were conducted using Wilcoxon rank-sum or Kruskal-Wallis tests as appropriate. Correlation analyses were performed using Spearman correlation coefficients. All statistical tests were two-sided, and p values less than 0.05 were considered statistically significant unless otherwise specified.

## Results

### Stemness-associated transcriptional alterations and prognostic signature construction in TCGA CRC

**Figure 1:**
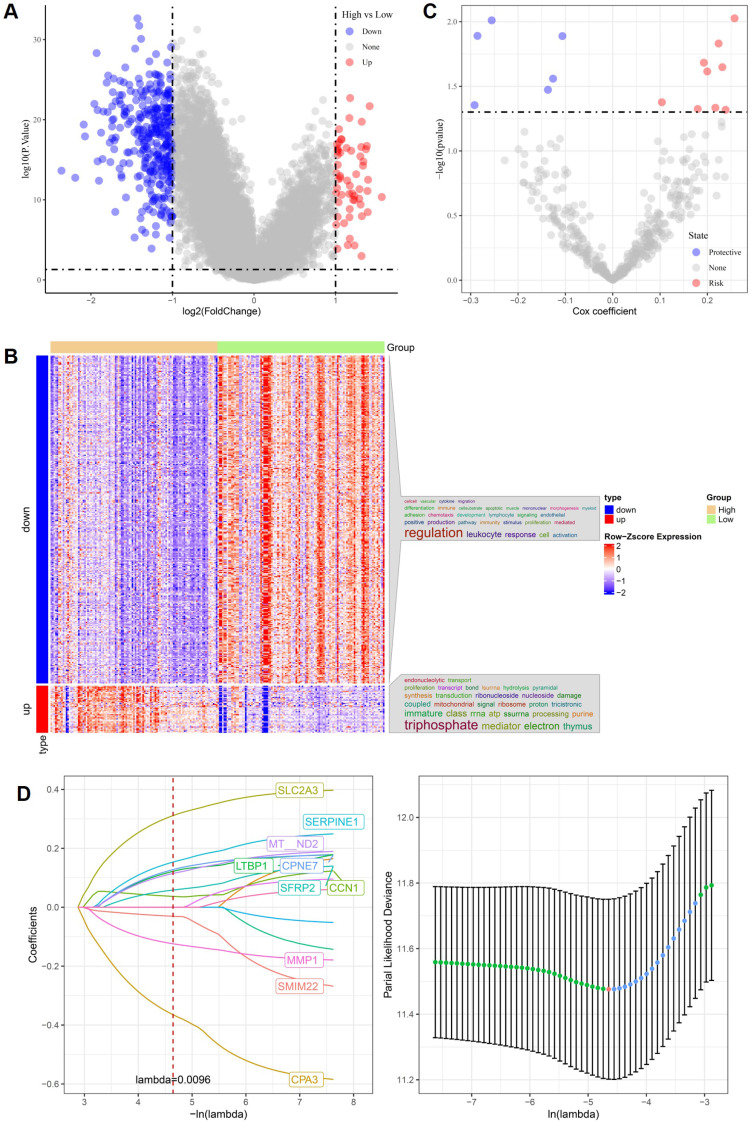
** Stemness-associated gene signatures and prognostic modeling in TCGA CRC.** (A) Volcano plot of differentially expressed genes between high- and low-stemness groups. (B) Heatmap of stemness-associated genes showing clear separation between stemness-defined tumors. (C) Univariate Cox regression analysis identifying risk-associated and protective genes. (D) LASSO Cox regression analysis demonstrating gene selection and optimal lambda determination.

Using bulk RNA-seq data from the TCGA CRC cohort, patients were stratified into high- and low-stemness groups based on transcriptome-derived stemness scores. Differential expression analysis revealed extensive stemness-associated transcriptional reprogramming, as illustrated by the volcano plot in Figure 1A. Genes upregulated in the high-stemness group and those downregulated exhibited clear asymmetrical distributions, indicating distinct molecular states associated with stemness in CRC. Hierarchical clustering of these differentially expressed genes demonstrated robust separation between high- and low-stemness tumors (Figure 1B). Genes enriched in high-stemness tumors were predominantly associated with immune-related and inflammatory processes, including leukocyte response and immune activation, whereas genes downregulated in the high-stemness group were mainly linked to mitochondrial function, ribosomal activity, RNA processing, and oxidative metabolism. This reciprocal pattern suggests that stemness-high CRC are characterized by immune-related transcriptional activation accompanied by metabolic suppression. To assess prognostic relevance, univariate Cox regression analysis was performed and identified multiple stemness-associated genes significantly correlated with overall survival (Figure 1C). Both risk-associated and protective genes were observed, indicating heterogeneous contributions of stemness-related pathways to patient outcomes. Subsequently, LASSO Cox regression was applied to construct a parsimonious prognostic model. An optimal lambda value of 0.0096 was determined by cross-validation, yielding a compact gene signature comprising SLC2A3, SERPINE1, MT-ND2, LTBP1, CPNE7, SFRP2, CCN1, MMP1, SMIM22, and CPA3 (Figure 1D). The stability of the cross-validation curve supported the robustness of this model. Overall, these results demonstrate that stemness-associated transcriptional programs identified from TCGA CRC data are tightly linked to immune regulation, metabolic remodeling, and clinical prognosis, providing a solid foundation for subsequent external validation and single-cell-level mechanistic analyses.

### Prognostic performance and external validation of the stemness-based risk model in CRC

**Figure 2:**
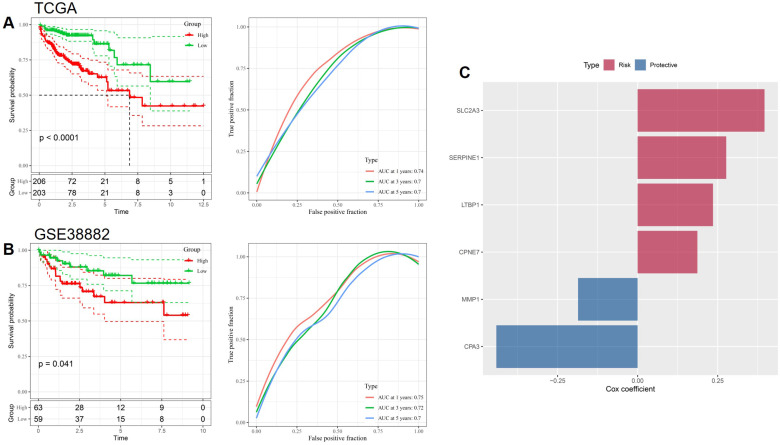
** Prognostic validation of the stemness-based risk model in CRC.** (A) Kaplan-Meier survival analysis and time-dependent ROC curves in the TCGA cohort. (B) External validation of survival discrimination and predictive performance in the GSE38882 cohort. (C) Cox regression coefficients of genes included in the prognostic signature, classified as risk-associated or protective.

To evaluate the prognostic value of the stemness-based gene signature, patients in the TCGA CRC cohort were stratified into high- and low-risk groups according to the calculated risk score. Kaplan-Meier survival analysis demonstrated that patients in the high-risk group exhibited significantly worse overall survival compared with those in the low-risk group (p < 0.0001, Figure 2A). Time-dependent ROC analysis further confirmed the predictive performance of the model, with AUC values of approximately 0.74, 0.70, and 0.70 at 1, 3, and 5 years, respectively, indicating stable prognostic accuracy over time. The robustness of the risk model was subsequently validated in an independent external cohort, GSE38882. Consistent with the TCGA results, high-risk patients in the validation cohort showed significantly poorer survival outcomes than low-risk patients (p = 0.041, Figure 2B). ROC analysis in GSE38882 yielded comparable AUC values across multiple time points, supporting the generalizability of the stemness-based signature across different patient populations and platforms. The contribution of individual genes to the prognostic model was further examined by Cox regression coefficients (Figure 2C). SLC2A3, SERPINE1, LTBP1, and CPNE7 were identified as risk-associated genes, whereas MMP1 and CPA3 functioned as protective factors. This balanced composition of risk and protective genes underscores the biological heterogeneity of stemness-associated pathways in CRC and supports the interpretability of the proposed prognostic model.

### Independent prognostic value and comparative performance of the stemness-based risk score in CRC

**Figure 3:**
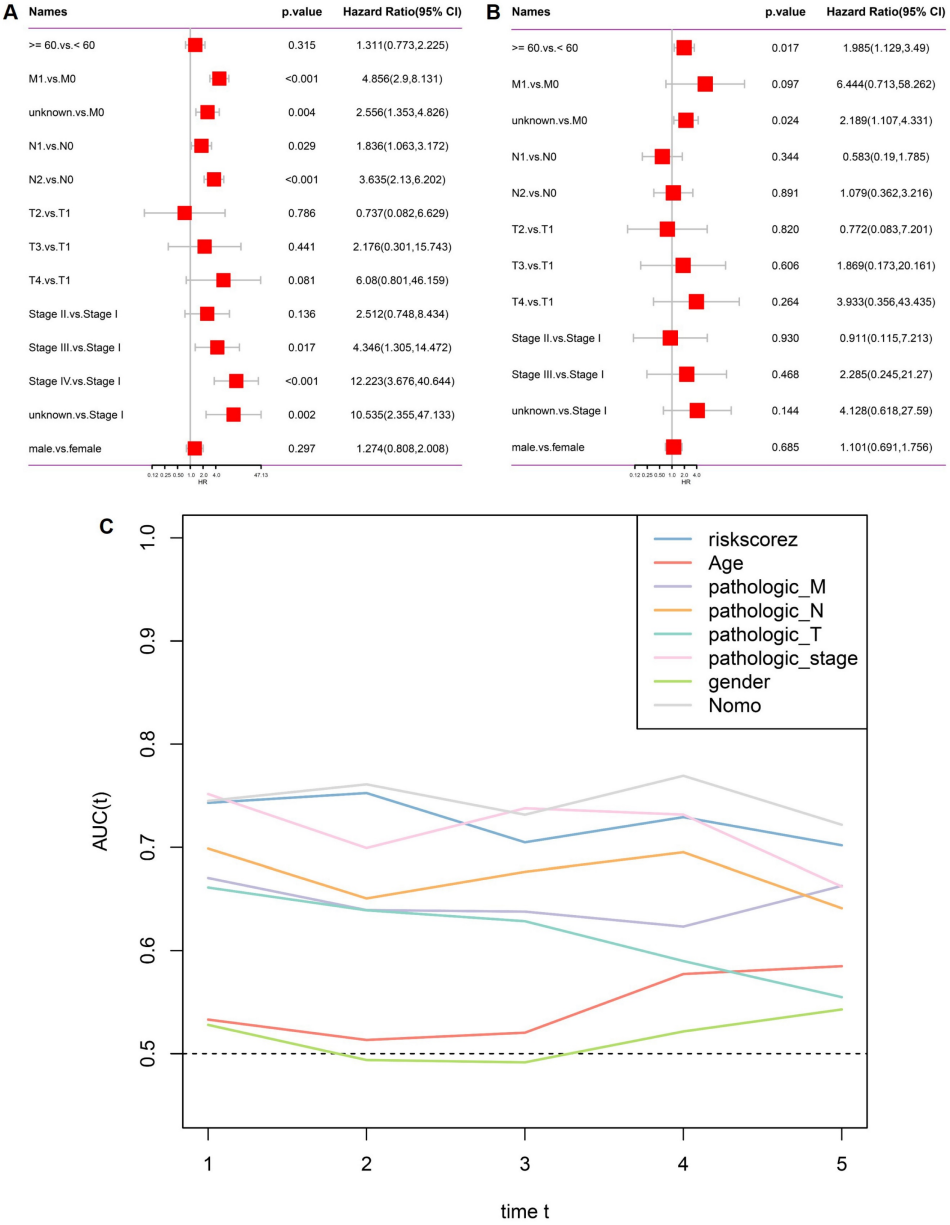
** Independent prognostic analysis and comparative predictive performance of the stemness-based risk model.** (A) Univariate Cox regression analysis of clinicopathological variables and risk score in the TCGA cohort. (B) Multivariate Cox regression analysis demonstrating the independent prognostic value of the risk score. (C) Time-dependent AUC comparison of the risk score, clinicopathological variables, and the integrated nomogram model.

To determine whether the stemness-based risk score serves as an independent prognostic factor, univariate and multivariate Cox regression analyses were performed in the TCGA CRC cohort. In univariate analysis, multiple clinicopathological variables, including metastatic status, nodal involvement, and advanced pathological stage, were significantly associated with overall survival, together with the risk score (Figure 3A). After adjustment for conventional clinical parameters, multivariate Cox regression demonstrated that the risk score remained independently associated with patient survival, whereas most clinicopathological features lost statistical significance (Figure 3B). These results indicate that the stemness-based risk model provides prognostic information beyond standard clinical staging variables. To further compare predictive performance, time-dependent AUC analysis was conducted. As shown in Figure 3C, the risk score consistently outperformed individual clinical factors, including age, pathological T, N, M status, and overall stage, across multiple time points. The integrated nomogram model achieved the highest AUC values, supporting the complementary value of combining molecular and clinical features for survival prediction in CRC.

### Association between the stemness-based risk score and the immune microenvironment in CRC

**Figure 4:**
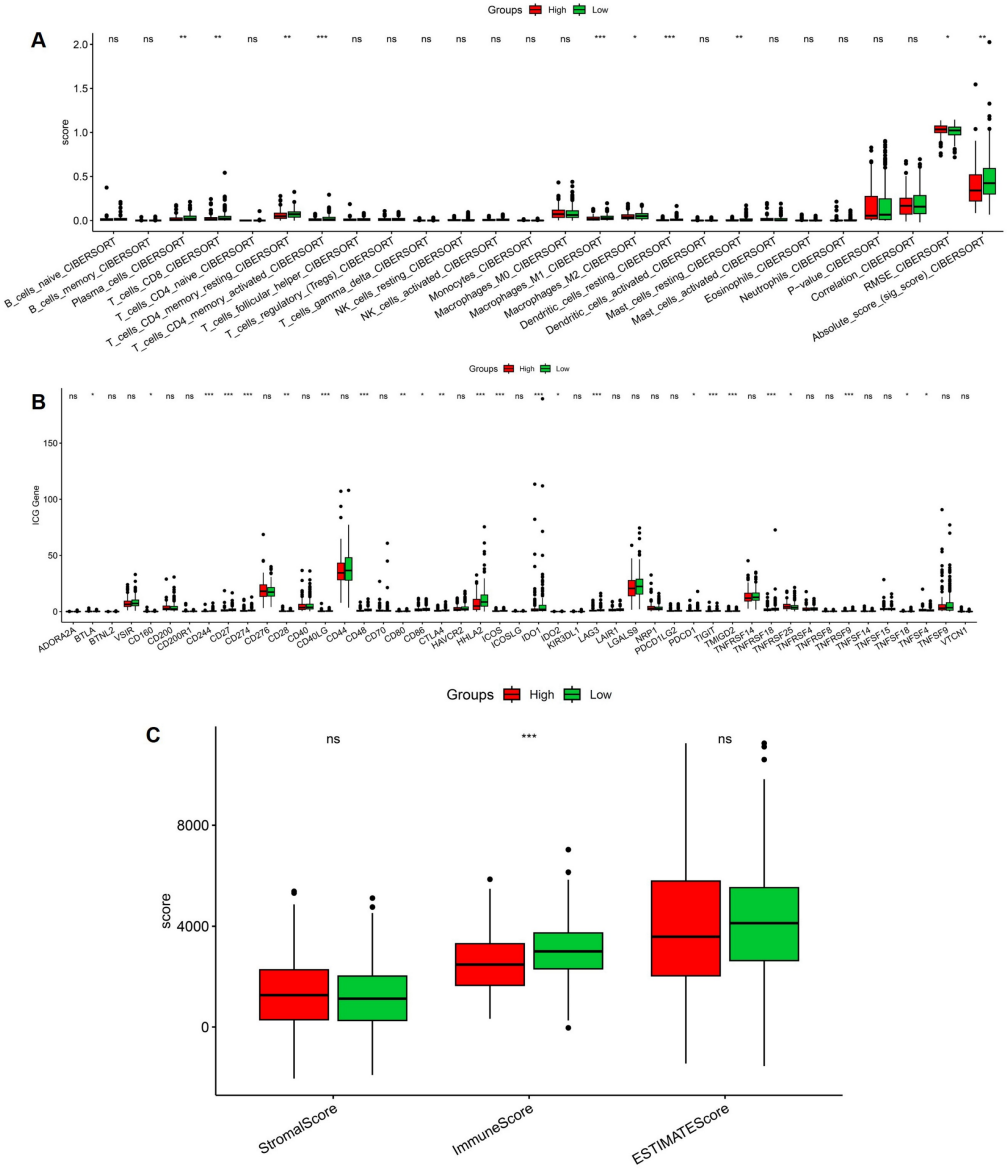
** Immune microenvironment characteristics associated with the stemness-based risk score in CRC.** (A) Comparison of CIBERSORT-estimated immune cell infiltration between high- and low-risk groups. (B) Differential expression of immune checkpoint-related genes between risk groups. (C) Comparison of stromal score, immune score, and ESTIMATE score between high- and low-risk tumors.

To explore the relationship between the stemness-based risk score and the tumor immune microenvironment, immune cell infiltration was estimated using CIBERSORT. Comparison between high- and low-risk groups revealed significant differences in multiple immune cell subsets, including activated T-cell populations, macrophage subtypes, and dendritic cells (Figure 4A). These findings indicate that the stemness-based risk stratification is accompanied by distinct immune infiltration patterns in CRC. We further examined the expression of immune checkpoint genes between risk groups. Several immune checkpoint-related genes exhibited differential expression between high- and low-risk tumors, suggesting altered immune regulatory states associated with stemness-related risk profiles (Figure 4B). This pattern is consistent with a more immunologically active yet potentially immunosuppressive microenvironment in high-risk CRCs. In addition, ESTIMATE analysis demonstrated that the high-risk group was associated with significantly higher immune scores, whereas stromal scores showed no significant difference between groups (Figure 4C). The ESTIMATE score, reflecting overall non-tumor cellular content, was also elevated in high-risk tumors. Collectively, these results suggest that the stemness-based risk score is closely linked to immune infiltration and immune regulatory activity in the colorectal tumor microenvironment.

### Clinical associations of stemness score in CRC

**Figure 5:**
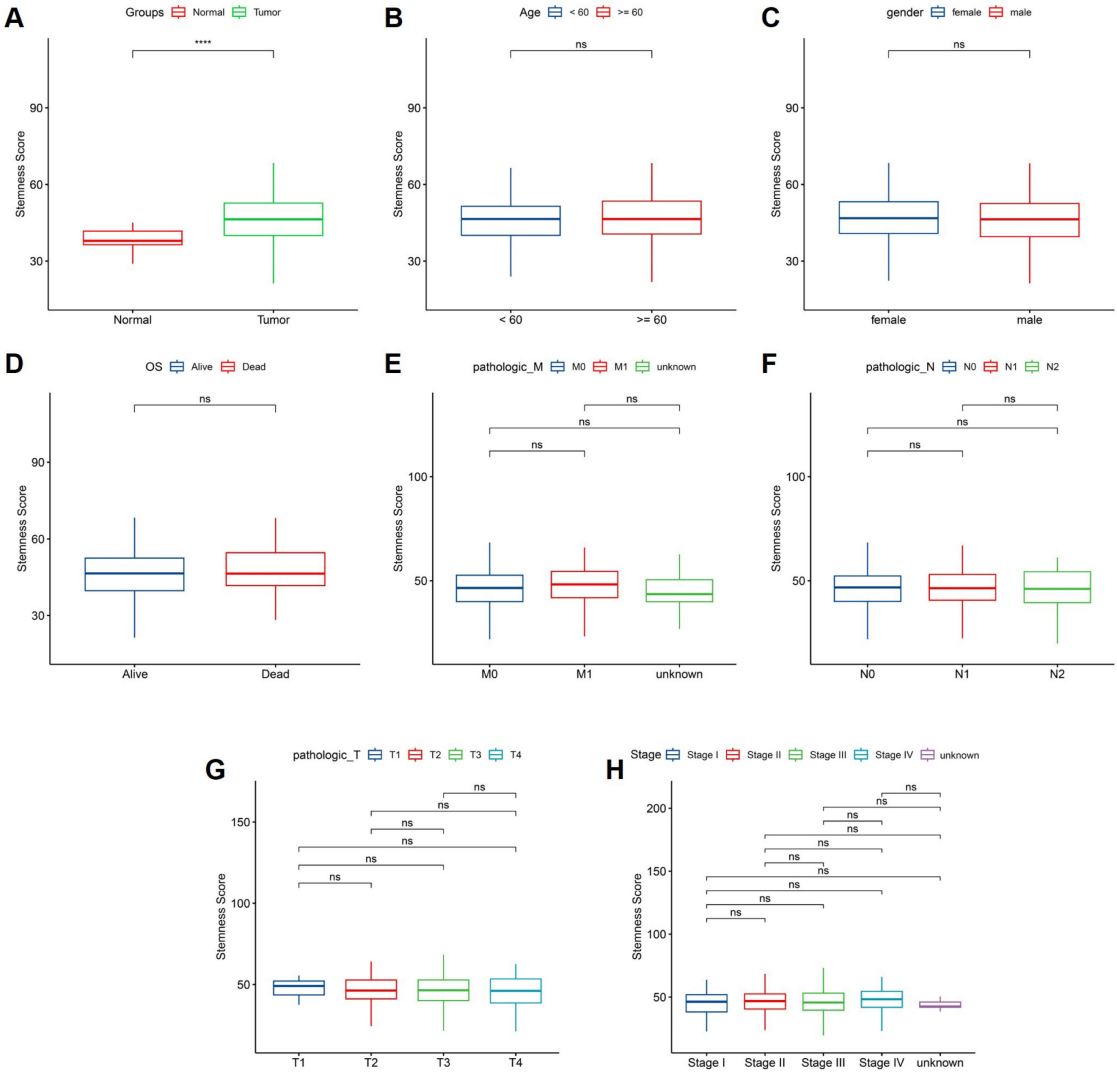
** Association between stemness score and clinicopathological features in CRC.** (A) Comparison of stemness score between normal and tumor tissues. (B-C) Stemness score stratified by age and sex. (D) Comparison of stemness score according to overall survival status. (E-H) Distribution of stemness score across pathological M, N, T classifications and tumor stage.

We next investigated the associations between stemness score and clinicopathological characteristics in the TCGA CRC cohort. As shown in Figure 5A, tumor tissues exhibited significantly higher stemness scores than normal tissues, indicating enhanced stemness features in CRC. In contrast, stemness scores did not differ significantly according to age or sex (Figure 5B-C). No significant difference in stemness score was observed between patients who were alive or deceased at the time of follow-up (Figure 5D). Similarly, stemness scores showed no significant associations with metastatic status, lymph node involvement, primary tumor depth, or overall pathological stage (Figure 5E-H). These results suggest that stemness score primarily reflects intrinsic tumor biological properties rather than conventional clinicopathological stratifications.

### Stemness score is tightly associated with immune checkpoint regulatory networks in CRC

**Figure 6:**
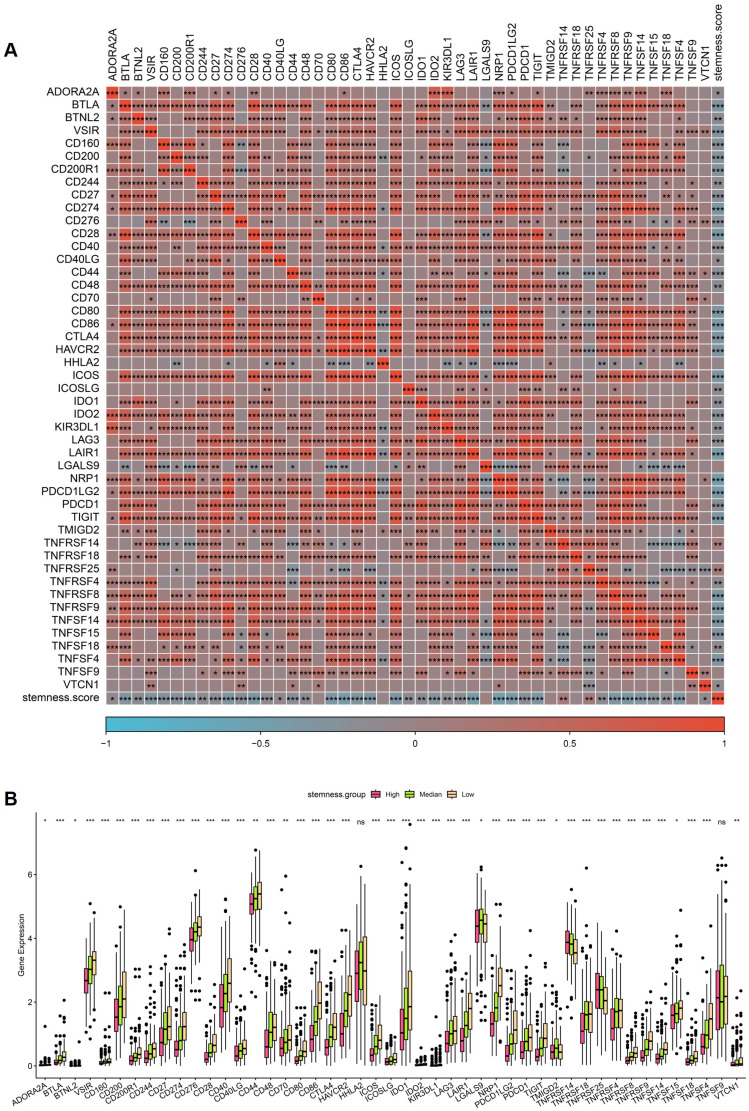
** Association between stemness score and immune checkpoint gene expression in CRC.** (A) Correlation heatmap showing relationships between stemness score and immune checkpoint-related genes in the TCGA cohort. (B) Comparison of immune checkpoint gene expression across high, median, and low stemness groups.

To further investigate the relationship between stemness and immune regulation, correlation analysis was performed between stemness score and immune checkpoint-related genes in the TCGA CRC cohort. As shown in Figure 6A, stemness score exhibited widespread positive correlations with multiple immune checkpoint genes, including PDCD1, CD274, CTLA4, LAG3, TIGIT, HAVCR2, IDO1, and members of the TNF and TNFR superfamilies. These results indicate that increased stemness is accompanied by coordinated activation of immune inhibitory signaling pathways. Patients were subsequently stratified into high, median, and low stemness groups to compare immune checkpoint gene expression levels. Consistent with the correlation analysis, the high-stemness group demonstrated significantly elevated expression of a broad range of immune checkpoint genes compared with the median and low stemness groups (Figure 6B). This stepwise increase in immune checkpoint expression across stemness strata suggests a stemness-dependent immunoregulatory gradient in CRC. Collectively, these findings support a close coupling between stemness-associated transcriptional programs and immune checkpoint activation, implying that stemness-high CRCs may adopt immune-evasive phenotypes through enhanced inhibitory immune signaling.

### Association between stemness score and immune evasion potential in CRC

**Figure 7:**
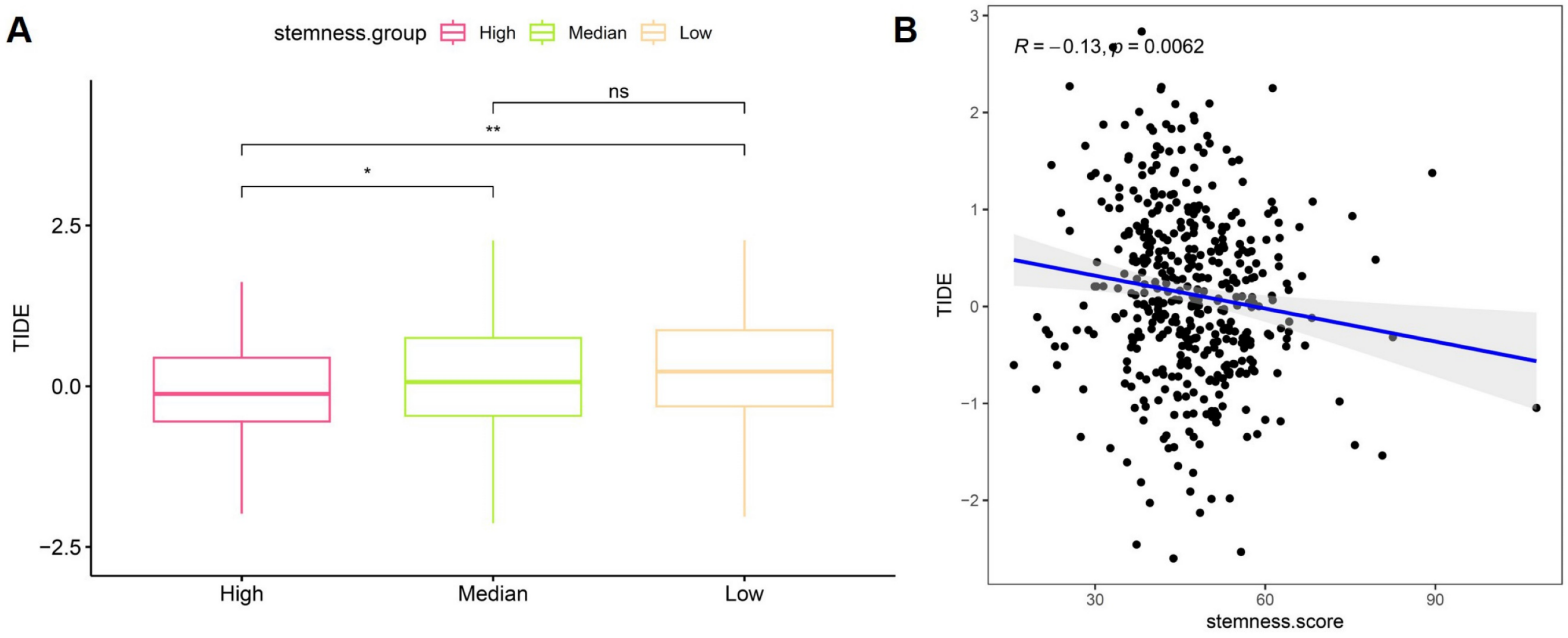
** Relationship between stemness score and immune evasion potential in CRC.** (A) Comparison of TIDE scores across high, median, and low stemness groups. (B) Correlation between stemness score and TIDE score in the TCGA cohort.

To evaluate the relationship between stemness and immune evasion, Tumor Immune Dysfunction and Exclusion (TIDE) scores were analyzed in the TCGA CRC cohort. Patients stratified by stemness levels showed significant differences in TIDE scores, with the high-stemness group exhibiting lower TIDE values compared with the median group, while no significant difference was observed between the median and low stemness groups (Figure 7A). Correlation analysis further demonstrated a modest but significant negative association between stemness score and TIDE score (R = -0.13, p = 0.0062), indicating that tumors with higher stemness tended to display reduced predicted immune escape potential (Figure 7B). These findings suggest that stemness-associated transcriptional programs are linked to immune modulation and may influence immunotherapy-related response patterns in CRC.

### Single-cell landscape of paired normal and tumor colorectal tissues reveals cell-type-specific transcriptional programs

**Figure 8:**
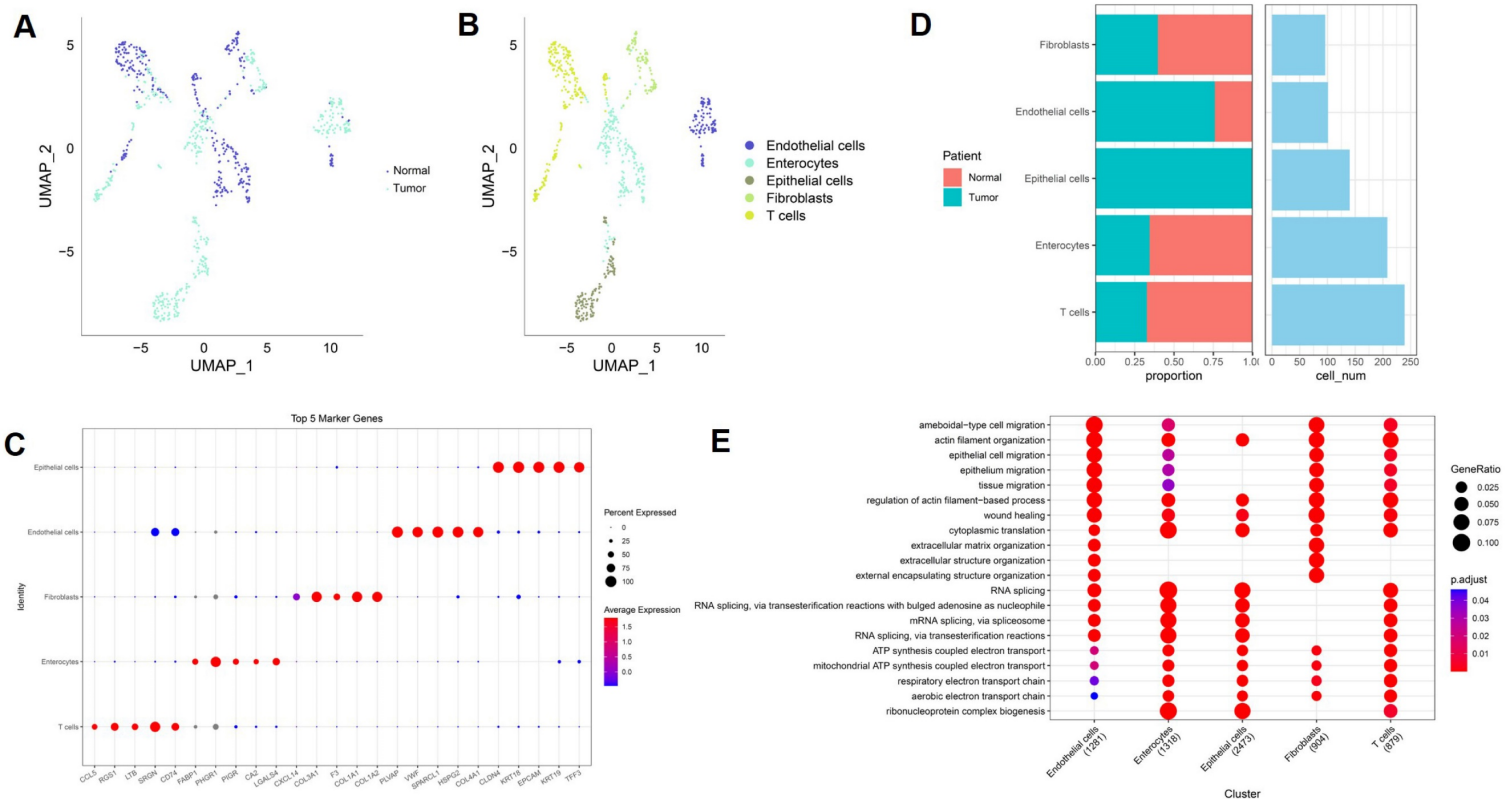
** Single-cell transcriptomic landscape of paired normal and tumor colorectal tissues in GSE196964.** (A) UMAP plot showing separation of normal and tumor cells. (B) UMAP visualization of annotated cell types. (C) Dot plot of representative marker genes across major cell populations. (D) Comparison of cell-type proportions and cell numbers between normal and tumor samples. (E) Functional enrichment analysis of major cell populations.

ScRNA-seq data from GSE196964 were analyzed to characterize the cellular composition of paired normal colon and colorectal tumor tissues. UMAP visualization demonstrated clear separation between normal and tumor cells, indicating distinct transcriptional states associated with malignant transformation (Figure 8A). Subsequent cell-type annotation identified five major cell populations, including epithelial cells, enterocytes, endothelial cells, fibroblasts, and T cells (Figure 8B). Marker gene analysis confirmed robust cell-type-specific expression patterns, with epithelial cells expressing EPCAM and KRT family genes, fibroblasts enriched for COL1A1 and COL1A2, endothelial cells expressing PECAM1 and VWF, enterocytes characterized by FABP1 and APOA1, and T cells marked by CD3D and CD3E (Figure 8C). These results validate the accuracy of cell-type annotation in this dataset. Quantitative comparison revealed marked differences in cellular composition between normal and tumor samples (Figure 8D). Tumor tissues exhibited an increased proportion of epithelial cells and fibroblasts, accompanied by a relative reduction in enterocytes and T cells, highlighting substantial remodeling of the tumor microenvironment in CRC. Functional enrichment analysis further revealed cell-type-specific biological processes (Figure 8E). Epithelial cells were predominantly enriched for RNA processing and metabolic pathways, fibroblasts showed strong enrichment in extracellular matrix organization and wound healing processes, endothelial cells were associated with migration and angiogenesis-related functions, and T cells displayed enrichment in immune activation-related pathways. Together, these findings delineate the multicellular architecture and functional heterogeneity of colorectal tumors at single-cell resolution.

### Single-cell CytoTRACE analysis reveals enhanced stemness and dedifferentiation in malignant colorectal cells

**Figure 9:**
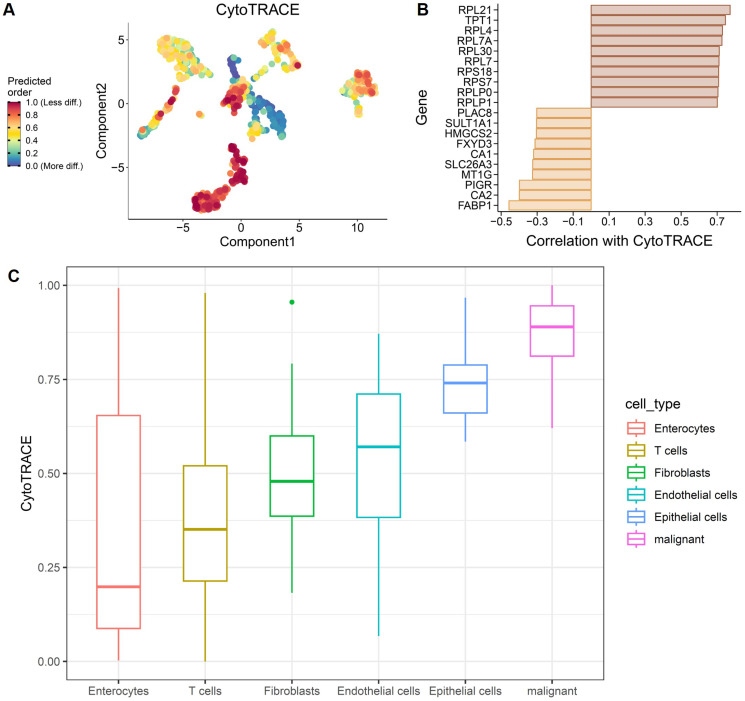
** CytoTRACE-based inference of differentiation states in CRC single-cell data.** (A) Projection of CytoTRACE scores onto low-dimensional embedding, indicating predicted differentiation states. (B) Genes positively and negatively correlated with CytoTRACE scores. (C) Distribution of CytoTRACE scores across major cell types.

To characterize cellular differentiation states at single-cell resolution, CytoTRACE analysis was applied to the GSE196964 dataset. Projection of CytoTRACE scores onto the low-dimensional embedding revealed a continuous gradient of predicted differentiation states across cell populations, with malignant epithelial cells exhibiting the highest CytoTRACE scores, indicative of reduced differentiation and enhanced stemness (Figure 9A). Correlation analysis identified genes positively and negatively associated with CytoTRACE scores (Figure 9B). Genes positively correlated with CytoTRACE were predominantly ribosomal and translational regulators, consistent with a progenitor-like transcriptional program, whereas genes negatively correlated with CytoTRACE included enterocyte differentiation markers such as FABP1, CA1, CA2, and SLC26A3, reflecting loss of mature intestinal epithelial features in stemness-high cells. Comparison of CytoTRACE scores across annotated cell types further confirmed a hierarchical differentiation landscape (Figure 9C). Malignant cells exhibited the highest CytoTRACE scores, followed by epithelial cells, endothelial cells, and fibroblasts, whereas enterocytes and T cells showed the lowest scores. These results demonstrate that colorectal tumor cells adopt a dedifferentiated, stemness-enriched state relative to normal epithelial and stromal compartments.

### Validation of the stemness-based risk model in independent immunotherapy cohorts

**Figure 10:**
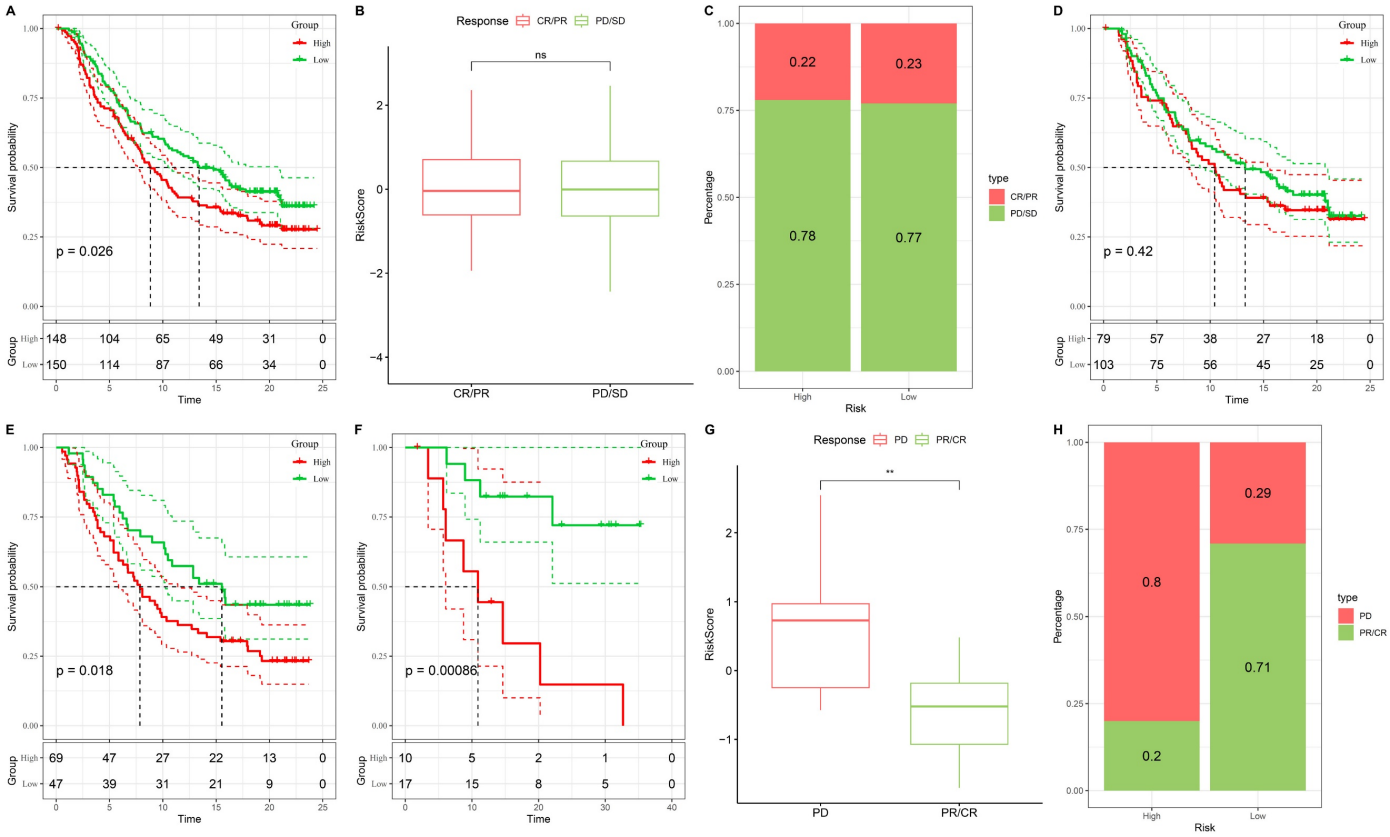
** Validation of the stemness-based risk model in immunotherapy datasets (IMvigor210 and GSE78820).** (A) Kaplan-Meier survival analysis of the IMvigor210 cohort showing significantly poorer survival in the high-risk group compared with the low-risk group. (B) Boxplot analysis in IMvigor210 demonstrating higher risk scores in patients with progressive disease or stable disease compared with those achieving complete or partial response. (C) Distribution of treatment responses in IMvigor210, with the low-risk group enriched for complete or partial response and the high-risk group enriched for progressive or stable disease. (D) Kaplan-Meier survival analysis of the GSE78820 cohort showing inferior survival outcomes in high-risk patients. (E) Subgroup Kaplan-Meier analysis in GSE78820 confirming a consistent trend toward reduced survival in the high-risk group. (F) Additional survival validation in a smaller GSE78820 subset further demonstrating poorer outcomes in high-risk patients. (G) Boxplot analysis in GSE78820 showing significantly higher risk scores in non-responders compared with responders. (H) Distribution of treatment responses in GSE78820 indicating that low-risk patients predominantly derived clinical benefit, whereas high-risk patients were mainly non-responders.

To validate the predictive value of the stemness-based risk model in the context of immunotherapy, two independent immunotherapy-treated cohorts, IMvigor210 and GSE78820, were analyzed. In the IMvigor210 cohort, Kaplan-Meier survival analysis demonstrated significantly poorer survival outcomes in patients classified into the high-risk group compared with those in the low-risk group (Figure 10A). Risk score distributions further revealed that patients with progressive disease or stable disease exhibited significantly higher risk scores than those achieving complete or partial response (Figure 10B). Consistently, response proportion analysis showed that the low-risk group was enriched for complete or partial responders, whereas the high-risk group was predominantly composed of patients with progressive or stable disease (Figure 10C).

These findings were independently confirmed in the GSE78820 cohort. High-risk patients displayed inferior survival outcomes compared with low-risk patients (Figure 10D), and subgroup survival analyses consistently demonstrated a trend toward reduced survival in the high-risk group across different validation settings (Figure 10E-F). In addition, non-responders in GSE78820 exhibited significantly higher risk scores than responders (Figure 10G). Response distribution analysis further indicated that most low-risk patients derived clinical benefit from immunotherapy, whereas high-risk patients were largely non-responders (Figure 10H). Collectively, these results demonstrate that the stemness-based risk model robustly predicts immunotherapy response and survival outcomes across independent cohorts, supporting its potential utility as a biomarker for immunotherapy stratification in CRC.

### Protein-level validation of stemness-associated signature genes in CRC tissues

**Figure 11:**
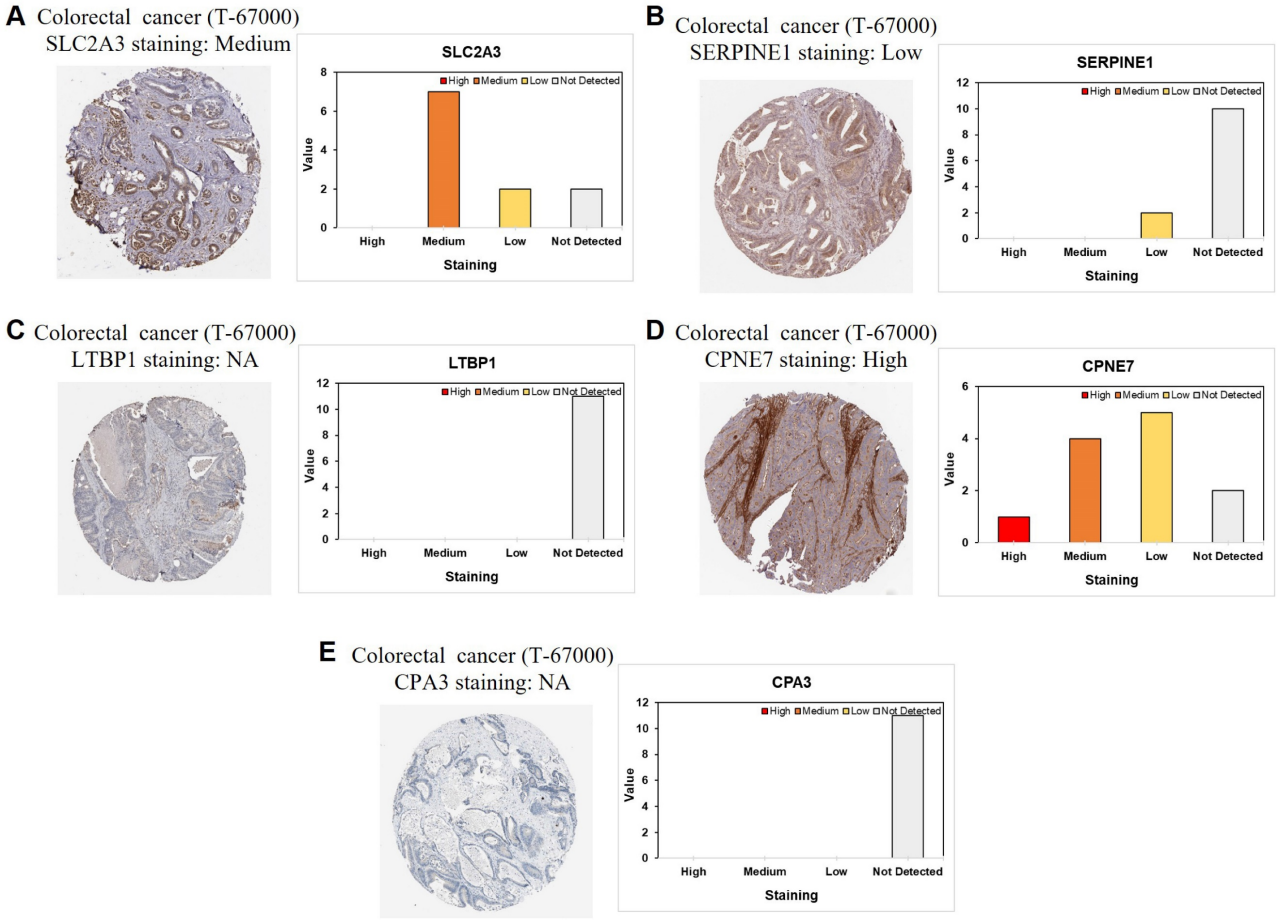
** Immunohistochemical validation of stemness-associated signature genes in CRC.** Representative immunohistochemical staining and corresponding intensity distribution of (A) SLC2A3, (B) SERPINE1, (C) LTBP1, (D) CPNE7, and (E) CPA3 in CRC tissue microarrays.

To further validate the stemness-associated risk signature at the protein level, immunohistochemical staining data from CRC tissue microarrays were examined. Representative staining patterns for SLC2A3, SERPINE1, LTBP1, CPNE7, and CPA3 are shown in Figure 11A-E. SLC2A3 protein expression exhibited predominantly medium staining intensity in CRC tissues, supporting its transcriptional upregulation observed in stemness-high tumors (Figure 11A). In contrast, SERPINE1 showed generally low or undetectable protein expression, suggesting potential post-transcriptional regulation or tumor heterogeneity affecting protein abundance (Figure 11B). LTBP1 and CPA3 proteins were largely undetectable in the analyzed samples, consistent with their variable or low expression patterns inferred from bulk and single-cell analyses (Figure 11C and 11E). Notably, CPNE7 demonstrated high protein expression in CRC tissues, aligning with its identification as a risk-associated gene in the prognostic model (Figure 11D). The differential protein expression patterns among these signature genes highlight heterogeneous regulatory mechanisms at the protein level and provide orthogonal validation for selected components of the stemness-based risk model. Collectively, these immunohistochemical findings support the biological relevance of the stemness-associated gene signature and reinforce its translational potential in CRC.

### Stemness-based risk score is associated with differential drug sensitivity in CRC

**Figure 12:**
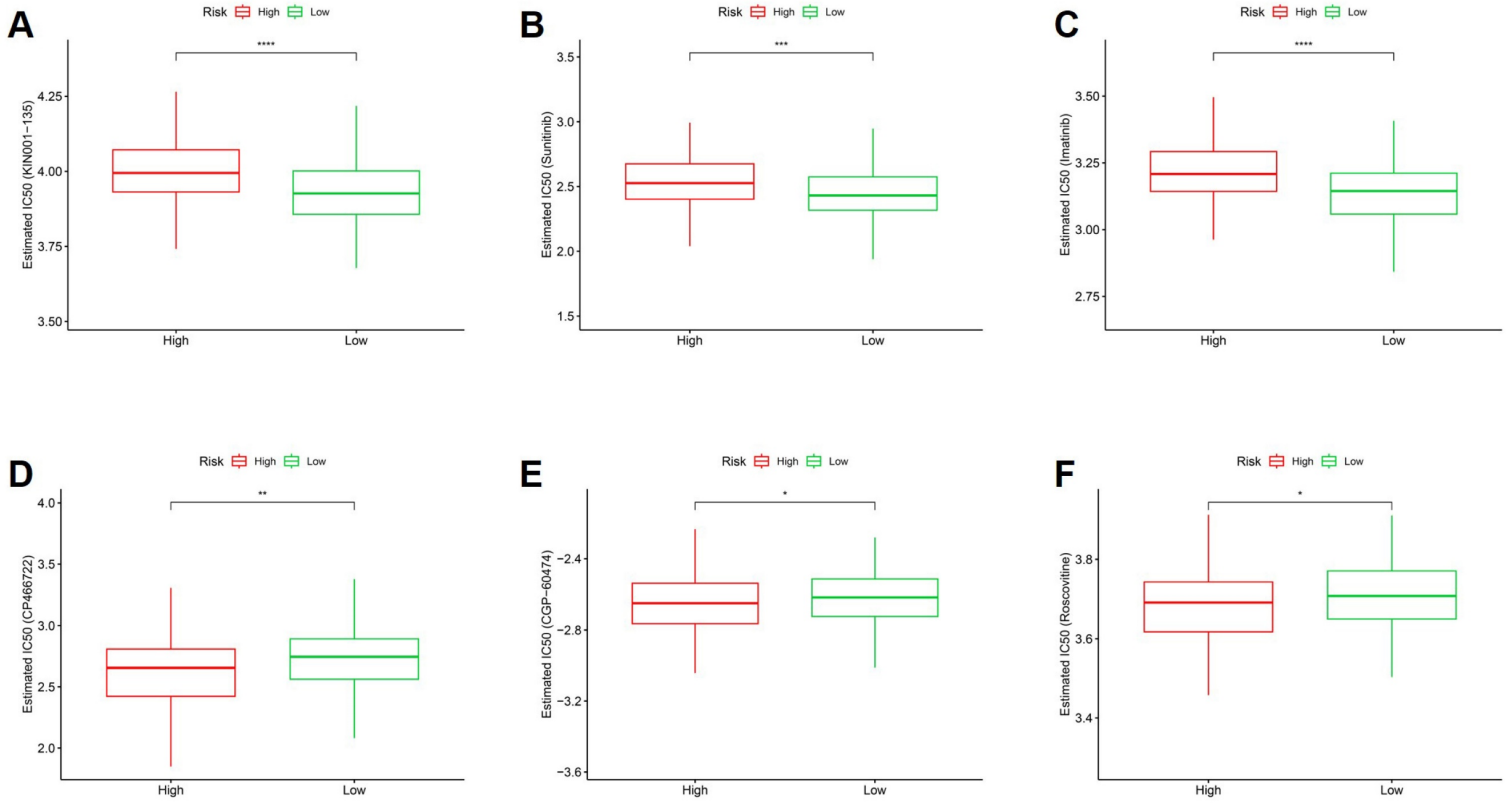
** Association between stemness-based risk groups and predicted drug sensitivity in CRC.** (A-C) Comparison of estimated IC50 values for KIN001-135, Sunitinib, and Imatinib between high- and low-risk groups. (D-F) Comparison of estimated IC50 values for CP466722, CGP-60474, and Roscovitine between risk groups.

These in silico IC50 estimations provide a global susceptibility profile, though they should be interpreted alongside the high degree of cellular heterogeneity. To explore the potential therapeutic implications of the stemness-based risk model, in silico drug sensitivity analysis was performed by comparing estimated IC50 values between high- and low-risk groups. As shown in Figure 12A-C, high-risk tumors exhibited significantly higher estimated IC50 values for multiple agents, including KIN001-135, Sunitinib, and Imatinib, indicating reduced drug sensitivity in stemness-high CRC. Conversely, low-risk tumors demonstrated significantly lower estimated IC50 values for several compounds, including CP466722, CGP-60474, and Roscovitine (Figure 12D-F), suggesting increased susceptibility to these agents. These differential drug response patterns indicate that stemness-associated risk stratification is closely linked to therapeutic vulnerability profiles. Collectively, these findings suggest that the stemness-based risk score may inform personalized treatment strategies by identifying subsets of CRC patients with distinct drug sensitivity landscapes. While providing a global profile, these estimations should be interpreted considering the inherent cellular heterogeneity identified in our single-cell analysis.

## Discussion

In this study, we systematically characterized the prognostic, immunological, and therapeutic relevance of tumor stemness in CRC by integrating bulk transcriptomic analyses, scRNA-seq, immunotherapy validation cohorts, and protein-level evidence. We developed a stemness-based risk model that robustly stratified patients into distinct prognostic groups across internal and external cohorts 17, 18. Beyond survival prediction, this model captured fundamental biological differences associated with stemness, including transcriptional reprogramming, immune microenvironment remodeling, and differential therapeutic vulnerabilities. These findings highlight tumor stemness as a central axis linking intrinsic tumor biology with clinical outcomes in CRC 69-71.

At the bulk transcriptomic level, stemness-associated gene expression patterns were strongly associated with adverse prognosis and remained independently predictive after adjustment for conventional clinicopathological variables. Importantly, the prognostic value of the stemness-based risk score exceeded that of individual clinical features and was further enhanced when integrated into a nomogram framework 72, 73. Immune profiling revealed that stemness-high tumors exhibited distinct immune infiltration patterns and coordinated upregulation of immune checkpoint-related genes, suggesting a complex immune regulatory state rather than simple immune exclusion 17, 74. Although the correlation between stemness score and TIDE score was modest, it was statistically significant and directionally consistent, which is expected given the biological heterogeneity of bulk data and the composite nature of immune evasion metrics. Crucially, the immunotherapy relevance of the stemness-based risk model was substantiated in two independent immunotherapy-treated cohorts, supporting its translational significance beyond correlation-based analyses 18, 75, 76.

By incorporating scRNA-seq data from paired normal and tumor colorectal tissues, we further resolved the cellular origins and differentiation states underlying stemness-associated phenotypes 26. Single-cell analysis demonstrated that malignant epithelial cells exhibited the highest stemness and dedifferentiation states, as inferred by CytoTRACE, while normal epithelial and immune cell populations showed more differentiated profiles 77. Functional enrichment analyses revealed that stemness-high malignant cells were characterized by transcriptional programs related to RNA processing, metabolic remodeling, and cellular plasticity, whereas stromal and endothelial compartments displayed distinct extracellular matrix and migratory signatures. These findings provide mechanistic support for the bulk-level observations and underscore the importance of cellular heterogeneity in shaping stemness-driven tumor behavior 78, 79.

From a therapeutic perspective, the stemness-based risk model was associated with differential sensitivity to multiple anticancer agents, suggesting potential utility for guiding personalized treatment strategies 69. In addition, protein-level validation using immunohistochemical data confirmed heterogeneous expression patterns of selected signature genes, reinforcing the biological relevance of the proposed model while also highlighting post-transcriptional and microenvironmental regulatory complexity 80. Nevertheless, several limitations should be acknowledged. The stemness score and risk model were derived primarily from retrospective datasets, and prospective clinical validation is warranted 17. Furthermore, while single-cell analyses provided valuable mechanistic insights, functional experiments will be required to establish causal relationships between stemness programs, immune modulation, and therapeutic response 69, 81.

In conclusion, our study presents an integrative framework that links tumor stemness with prognosis, immune regulation, and therapeutic vulnerability in CRC. By combining bulk and single-cell transcriptomics with immunotherapy validation and protein-level evidence, we provide a comprehensive view of stemness as a key determinant of tumor heterogeneity and clinical outcome. These findings lay the groundwork for future studies aimed at targeting stemness-associated pathways and optimizing precision oncology strategies in CRC. This independence suggests that stemness features may represent an intrinsic, early-stage molecular event that precedes anatomical progression.

## Conclusions

In this study, we established a stemness-based risk model that effectively stratifies CRC patients according to prognosis and therapeutic relevance. By integrating bulk transcriptomic analyses with scRNA-seq, immunotherapy validation cohorts, and protein-level evidence, we demonstrate that tumor stemness is a key determinant of tumor heterogeneity, immune regulation, and clinical outcome in CRC. Our findings indicate that stemness-associated transcriptional programs are linked to adverse survival, distinct immune microenvironment characteristics, and differential sensitivity to immunotherapy and anticancer agents. Single-cell analyses further revealed that enhanced stemness and dedifferentiation are predominantly localized within malignant epithelial cells, providing mechanistic support for bulk-level observations. Collectively, this integrative framework highlights the potential of stemness-based biomarkers to inform prognostic assessment and therapeutic stratification in CRC, and provides a foundation for future studies aimed at translating stemness-targeted strategies into precision oncology applications.

## Data Availability

All datasets and materials generated in this study can be provided by the corresponding author upon reasonable request.

## Abbreviations

CRC: colorectal cancer
TCGA: The Cancer Genome Atlas
GEO: Gene Expression Omnibus
OS: overall survival
ROC: receiver operating characteristic
AUC: area under the curve
HR: hazard ratio
CI: confidence interval
LASSO: least absolute shrinkage and selection operator
ssGSEA: single-sample gene set enrichment analysis
TME: tumor microenvironment
IHC: immunohistochemistry
scRNA-seq: single-cell RNA sequencing
UMAP: uniform manifold approximation and projection
PCA: principal component analysis
DEGs: differentially expressed genes
GO: Gene Ontology
KEGG: Kyoto Encyclopedia of Genes and Genomes
CIBERSORT: cell-type identification by estimating relative subsets of RNA transcripts
ESTIMATE: estimation of stromal and immune cells in malignant tumor tissues using expression data
TIDE: tumor immune dysfunction and exclusion
IC50: half-maximal inhibitory concentration
PR: partial response
CR: complete response
SD: stable disease
PD: progressive disease
KM: Kaplan-Meier
TMA: tissue microarray
CytoTRACE: cellular trajectory reconstruction analysis using gene counts and expression
